# Preparation and Characterization of Amorphous Solid Dispersions for the Solubilization of Fenretinide

**DOI:** 10.3390/ph16030388

**Published:** 2023-03-02

**Authors:** Guendalina Zuccari, Eleonora Russo, Carla Villa, Alessia Zorzoli, Danilo Marimpietri, Leonardo Marchitto, Silvana Alfei

**Affiliations:** 1 Department of Pharmacy (DiFAR), University of Genoa, Viale Cembrano 4, I-16148 Genova, Italy; 2 Cell Factory, IRCCS Istituto Giannina Gaslini, Via Gerolamo Gaslini 5, 16147 Genova, Italy; 3 Department of Sciences for the Quality of Life, University of Bologna, Corso D’Augusto 237, 47921 Rimini, Italy

**Keywords:** Fenretinide, neuroblastoma, drug delivery, nanoparticles, co-precipitation, lipophilic drugs, solubilization

## Abstract

Fenretinide (4-HPR), a retinoid derivative, has shown high antitumor activity, a low toxicological profile, and no induction of resistance. Despite these favorable features, the variability in oral absorption due to its low solubility combined with the high hepatic first pass effect strongly reduce clinical outcomes. To overcome the solubility and dissolution challenges of poorly water-soluble 4-HPR, we prepared a solid dispersion of the drug (4-HPR-P5) using a hydrophilic copolymer (P5) previously synthesized by our team as the solubilizing agent. The molecularly dispersed drug was obtained by antisolvent co-precipitation, an easy and up-scalable technique. A higher drug apparent solubility (1134-fold increase) and a markedly faster dissolution were obtained. In water, the colloidal dispersion showed a mean hydrodynamic diameter of 249 nm and positive zeta potential (+41.3 mV), confirming the suitability of the formulation for intravenous administration. The solid nanoparticles were also characterized by a high drug payload (37%), as was also evidenced by a chemometric-assisted Fourier transform infrared spectroscopy (FTIR) investigation. The 4-HPR-P5 exhibited antiproliferative activity, with IC50 values of 1.25 and 1.93 µM on IMR-32 and SH-SY5Y neuroblastoma cells, respectively. Our data confirmed that the 4-HPR-P5 formulation developed herein was able to increase drug apparent aqueous solubility and provide an extended release over time, thus suggesting that it represents an efficient approach to improve 4-HPR bioavailability.

## 1. Introduction

Fenretinide (N-(4-hydroxyphenyl)retinamide (4-HPR), is a synthetic derivative of retinoic acid (RA), belonging to the third generation of retinoids, first synthesized in the late 1960s [1]. It immediately attracted the attention of the scientific community, since in early in vitro studies it showed an antitumor efficacy against a wide range of cancers at concentrations ranging from 1 to 10 µM [2]. In addition, its mechanisms of action appeared quite different from its natural congener, all-*trans*-retinoic acid (ATRA). Indeed, 4-HPR does not promote either cellular differentiation or the emergence of resistance. On the contrary, it is endowed with a more favorable toxicological profile and a better tissue distribution than previous retinoids and is successful as a chemo preventive agent in the treatment of breast and ovarian cancer [3]. Therefore, thanks to its effects on both premalignant cells and transformed cells, 4-HPR has been extensively studied for further development in clinical applications. Unfortunately, clinical trials have often shown high variability in their results, mainly due to the massive hepatic first pass effect and the very poor water solubility of 4-HPR (log P_W/O_ = 6.3), which constrain the plasmatic concentration of 4-HPR to below an effective level. The first clinical trials employed oral gelatin capsules containing 100 mg 4-HPR solubilized in corn oil and polysorbate 80. The low bioavailability, estimated at 16%, and the poor patient compliance in taking the required number of capsules strongly limited the effectiveness of this strategy [4]. As a result, new oral and parenteral formulations are required to enhance the bioavailability of 4-HPR and assure steady-state plasma concentrations near 10 µM. To date, several attempts have been made, including an oral formulation composed of a mixture of monoglycerides, free fatty acids, and lysophosphatidylcholine, called LYM-X-SORB™. It was administered in combination with sugar to mask the bitter taste of the waxy lipid matrix at doses ranging from 352 to 2210 mg/m^2^ (divided twice daily) for seven days every three weeks. The 4-HPR mean peak plasma levels reached 21 µM at 1700 mg/m^2^/day on day 6 of cycle 1. Out of 29 patients, four had complete responses, while six had a stable disease, confirming the significant interpatient variability with oral dosing [5].

Thus, while 4-HPR has potentially novel mechanisms of anticancer activity and a reduced systemic toxicity, its poor aqueous solubility represents a significant challenge to achieving adequate tumor concentrations, which are required to optimize its anticancer activity. Following these considerations, intravenous formulations were also developed, such as a lipid emulsion containing egg phospholipids, glycerin, alcohol, and soybean oil. The formulation was infused in continuo at 905–1414 mg/m^2^/day for 5 days in 21-day cycles as part of a phase I study involving 23 patients. Nevertheless, although 4-HPR plasma steady-state concentrations were in the range of 17.87–38 µM, no patients had objective responses, while five patients showed stable disease as the best response (28%) [6]. 

In recent years, many other techniques have been employed attempting to enhance the drug aqueous solubility, including micellar solubilization with amphiphilic dextrins or branched polyethylene glycol [7,8], conjugation with polyvinyl alcohol [9], encapsulation into PLGA microparticles [10], liposomes [11], (recently) drug ionization [12], and amorphization [13,14]. Drug amorphization, in addition to enhancing the drug solubility and dissolution rate, provokes the generation of a supersaturated solution, which creates a higher flux across the intestinal membrane [15]. Unfortunately, the amorphous state is unstable, thus tending to revert to the less soluble crystalline form, with quality and efficacy issues. A strategy to inhibit crystallization consists of formulating the drug with a polymer, thus forming an amorphous solid co-dispersion (ASD) [16]. In the pharmaceutical industry, several processing approaches have been employed to prepare amorphous materials, mainly including hot-melt extrusion and spray drying [17,18,19,20,21,22]. However, novel processing technologies have emerged to meet the growing physicochemical complexities of the modern pharmaceutical pipeline [17]. Antisolvent co-precipitation is one such emerging manufacturing approach, notable for its ability to allow the production of ASDs containing thermally instable and water-insoluble pharmaceuticals, demonstrating improved mechanical and material properties compared to ASDs generated by more conventional methods [17]. It involves the precipitation of drug particles within a polymeric matrix used as solubilizing agent from a supersaturated solution of the two ingredients. This approach could simultaneously achieve the goals of drug amorphization and nanosizing. By employing only amorphous polymers to molecularly dissolve or disperse the drug via co-precipitation, second-generation ASDs (SG) are achieved; by adding a surfactant to the system to increase the drug dissolution and reduce the problems of precipitation and recrystallization, third-generation ASDs (TG) are obtained [23]. To our knowledge, the antisolvent co-precipitation process is a technique that has not yet been explored to improve the physicochemical properties of 4-HPR. Thus, herein, we propose a nanoprecipitation technique for the preparation of 4-HPR-loaded nanoparticles (4-HPR-P5 NPs) employing a water-soluble cationic copolymer (P5) obtained by copolymerizing the laboratory-made monomer 4-ammoniumbuthylstyrene hydrochloride with di-methyl-acrylamide (DMAA) as an uncharged diluent [24]. P5 was found to be able to significantly increase the production of reactive oxygen species (ROS) at 2.5 µM and reduce the neuroblastoma (NB) cell viability of both etoposide-sensitive and etoposide-resistant HTLA-230 NB cells in a dose-dependent manner [25]. Therefore, P5 seemed to us a promising candidate to increase the apparent solubility of 4-HPR, thanks to its hydrophilicity, and simultaneously exert a possible synergistic antitumor effect. In this study, we prepared and characterized 4-HPR-P5 NPs, and the formulation was tested for its cytotoxic activity against neuroblastoma cell lines.

## 2. Results and Discussion

### 2.1. Preparation of 4-HPR-P5 NPs

**Scheme 1:**
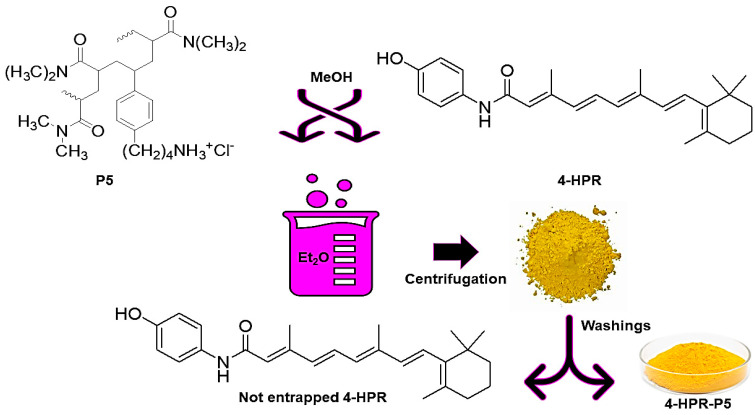
Synthetic pathway to prepare 4-HPR-P5 NPs.

An efficient entrapment of bioactive molecules in polymers, copolymers, and dendrimer nanoparticles can improve their solubility, stability, and efficacy, as well as reduce their toxicity [26]. In this regard, with 4-HPR being scarcely soluble in water and chemically instable when exposed to light and heat, its encapsulation in highly water-soluble P5 could allow an enhancement in its water solubility while protecting it from early degradation. Additionally, in cases of a high drug-loading capacity (DL%), several equivalents of the entrapped 4-HPR could be released at the target site upon the administration of a very low dosage of the complex P5/4-HPR. Finally, since P5 has been shown to possess a remarkable ROS-dependent cytotoxic effect against neuroblastoma cells both sensitive (4.3 µM) and resistant (2.2 µM) to etoposide [25], a possible synergistic effect was hypothesized. Based on these considerations, 4-HPR was entrapped in the cationic copolymer P5, as previously reported [25]. To prepare our 4-HPR-loaded polymeric NPs, we considered the co-precipitation process as the simplest and most reproducible method [26]; therefore, 4-HPR-P5 NPs were prepared according to Scheme 1.

**Figure 1:**
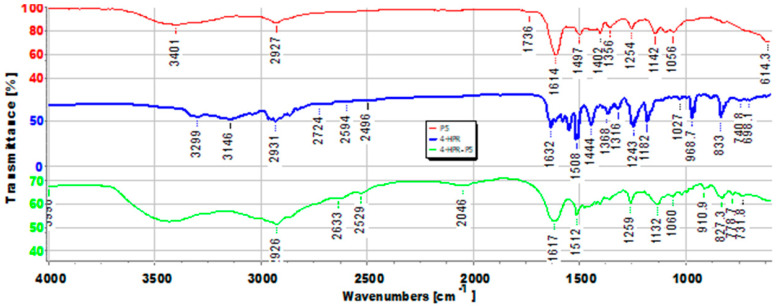
FTIR spectra of 4-HPR-P5 NPs (green line), P5 (red line), and pristine 4-HPR (blue line) for easier comparison.

Over the last few decades, antisolvent precipitation, as a way of generating supersaturation, has drawn increasing attention in the pharmaceutical field. Indeed, precipitation techniques are relatively simple, low-cost, and easily up-scalable, as they can be carried out in a continuous fashion using static mixers [27]. Usually, an aqueous phase is used as an antisolvent to precipitate the drug/polymer complex. In our case, since P5 was soluble in water, the aqueous phase was replaced by Et_2_O, in which P5 was insoluble and 4-HPR poorly soluble. MeOH was chosen to solubilize both P5 and 4-HPR, producing a clear yellow methanol solution. The addition of the methanol solution of P5 and 4-HPR to the non-solvent under moderate stirring led to an abrupt and simultaneous nucleation, which was ideal for obtaining small and uniform nanocrystals [28]. Moreover, by adding the “good solvent” solution of the drug to the antisolvent solution, and not the contrary, we could avoid the occurrence of localized supersaturation during the mixing process, which may have activated successive nucleation and yielded fewer and larger crystals [28]. Finally, purified 4-HPR-P5 NPs were obtained as a yellow solid, which was stored at −18 °C in the dark. From the organic solvents used for the reactions and ethereal washings, unentrapped 4-HPR was recovered, and its identity was confirmed by FTIR analysis (not reported), TLC, and PCA. Figure 1 shows the FTIR spectrum of the 4-HPR-P5 NPs, wherein bands belonging to both P5 (2926, 1617, 1259, 1132, and 1060 cm^−1^) and the entrapped 4-HPR (2633, 2529, 1512, and 827 cm^−1^) could be detected, thus confirming the successful loading.

### 2.2. Principal Component Analysis (PCA) of ATR-FTIR Data

**Figure 2:**
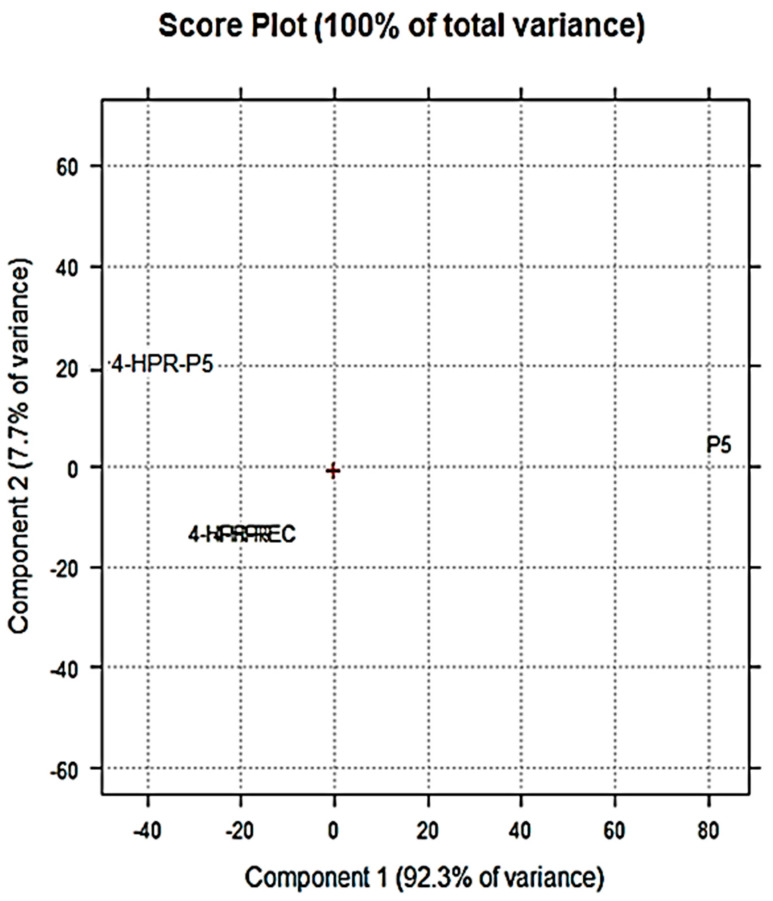
PCA results, represented as a score plot (PC1 vs. PC2).

To further confirm the presence of 4-HPR in the prepared NPs, we processed the FTIR spectral data using PCA [29,30], which allowed us to visualize the reciprocal positions occupied by the 4-HPR, P5, 4-HPR-P5 NPs, and recovered unentrapped 4-HPR (4-HPR-R) in the score plot of PC1 (explaining 92.3% of the variance) vs. PC2 (explaining 7.7% of the variance) (Figure 2).

Except for 4-HPR and 4-HPR-R, which had identical scores and thus confirmed that the substance recovered from the solvents was 4-HPR unentrapped in P5, the samples were well-separated on PC1 and reciprocally located at score values. This showed that the prepared NPs were structurally more like 4-HPR than P5, thus demonstrating the high content of 4-HPR in the prepared NPs. In particular, while P5 was located at positive scores on PC1, NPs containing 4-HRP, 4HRP, and 4-HRP-R were all located at negative scores.

### 2.3. Potentiometric Titrations of 4-HPR-P5 NPs

**Figure 3:**
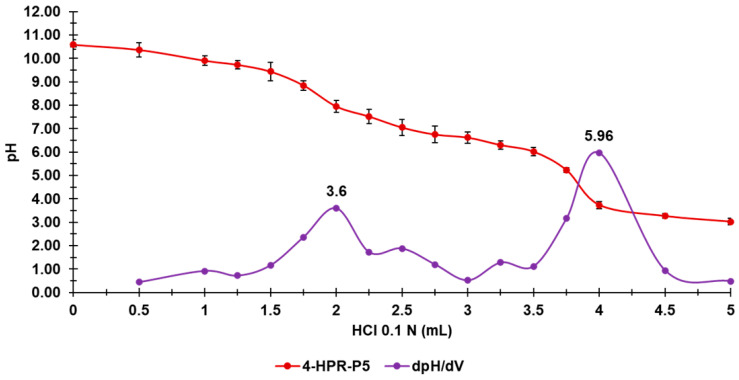
Potentiometric titration of 4-HPR-P5 (red line); first derivative (dpH/dV) of the titration curve (purple line).

The titration curve of 4-HPR-P5 was obtained by plotting the measured pH values vs. the aliquots of HCl 0.1N added (Figure 3, red line). Subsequently, from the titration data, the dpH/dV values were computed and reported in the same graph vs. those of the corresponding volumes of HCl 0.1N, thus generating the first derivative line of the titration curve (Figure 3, purple line), whose maxima represent the titration end points (or the various phases of the protonation process).

As is observable in Figure 3, 4-HPR-P5 showed a two-phase protonation process corresponding to two maxima in the first derivative curve. The first was observed upon the addition of 2 mL of HCl 0.1 N (pH = 7.95), while the second, representing the titration end point, was observed when 4 mL of HCl 0.1 N (pH = 3.74) was added. The potentiometric titration of 4-HPR-P5 NPs was useful to determine their buffer capacity (β = dV/dpH) and their average buffer capacity (β_AVE_). It has been extensively reported that cationic materials, such as those used to transport genetic material inside cells for gene-therapy purposes, are also internalized by endocytosis [25,31,32]. Upon endocytosis, the internalized material is incorporated into an endosome, which is promptly assailed and degraded by lysosomes if a rapid escape does not occur. In cationic gene-delivery systems, this goal is achieved by the implementation of a “proton sponge” that attracts the protons inside the endosome, thus causing its osmotic swelling and bursting [25]. The proton sponge activity of cationic macromolecules mainly depends on their buffer capacity (β = dV(HCl)/dpH)) [33], particularly in the pH range 7.4–5.1, and on their average buffer capacity (β_AVE_ = dV(HCl)/dpH(1)), which is the mean of the volume of HCl necessary to decrease the pH by one unit in the pH range 4.5–7.5 [34]. The proton sponge activity improves with an increase in the values of β and β_AVE_. Thus, to predict the ability of 4-HPR-P5 NPs to avoid premature degradation and inactivation, we evaluated their β and β_AVE_. The potentiometric titration data were exploited to compute β and β_AVE_ according to the abovementioned formulas in brackets (pH range 4.5–7.5). The maximum β value observed for 4-HPR-P5 and its value of β_AVE_ are reported in Table 1 and compared with those previously obtained for commercial branched PEI-*b* (25 kDa), a reference standard recognized for its good buffer capacity, and empty P5 [25].

**Figure 4:**
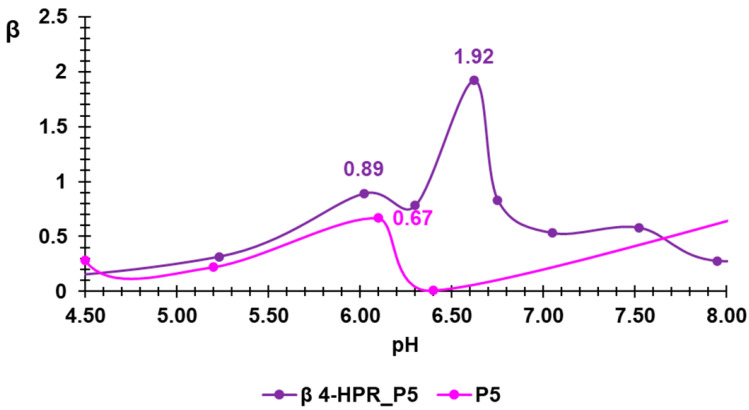
Buffer capacity curves of P5 and 4-HPR-P5 NPs.

By plotting the β values determined for P5 and 4-HPR-P5 NPs in the desired pH range vs. the values of the corresponding pH, their buffer capacity curves were obtained, which clearly showed the maximum values reported in Table 1 (Figure 4).

**Figure 5:**
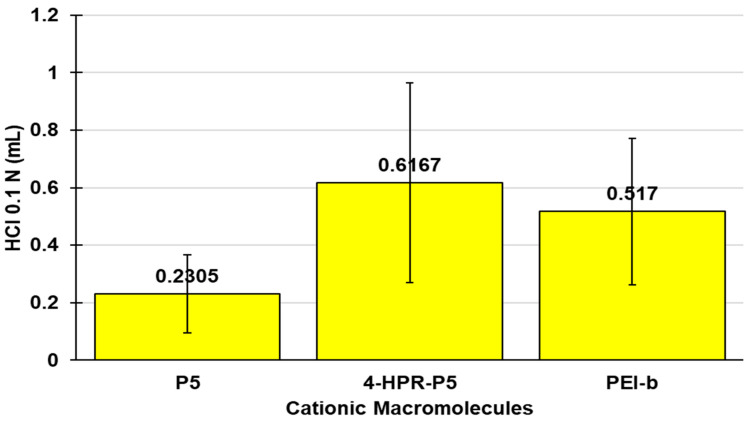
Average buffer capacity of P5, 4-HPR-P5, and PEI-*b* 25 kD.

As reported in Table 1, PEI-*b* showed two maxima in the pH range of interest, at pH values of 6.81 and 7.33, which were about ten times lower than that of P5 and several times lower than that of 4-HPR-loaded P5-based NPs; hence, the β curve of PEI is not reported in Figure 4. These findings established that, at fixed pH points within the pH range of interest, both P5 and 4-HPR-P5 NPs possessed a buffer capacity far higher than that of PEI-*b*. As is observable in Figure 4, upon the entrapment of 4-HPR, the buffer capacity of P5 was further increased and shifted towards higher pH values. Interestingly, although P5 demonstrated a β value higher than that of PEI-*b*, it had a lower β_AVE_ value, as is also observable in Figure 5. On the contrary, 4-HRP-P5 NPs demonstrated values of both β and β_AVE_ higher than those of PEI-*b*.

### 2.4. 4-HPR-P5 NPs Characterization

Although there have been many studies on antisolvent precipitation, its mechanism is poorly understood compared to other methods [27]. The effectiveness of precipitation was high, since starting from about 200 mg of raw materials we obtained 163 mg of solid 4-HPR-P5 NPs (79%), confirming the suitability of the solvent selected to induce supersaturation. The DL %, determined by UV-Vis spectrophotometric analysis using a previously constructed linear calibration curve available in the Supplementary Information (SI) (Figure S1), was found to be 37 ± 3.46%. The value was notably high and suggested the formation of strong interactions between the drug and the copolymer and their repulsion for the antisolvent. This high drug content could exert a negative impact on the aqueous solubility of loaded NPs; however, the total drug solubility actually estimated from the 4-HPR-P5 saturated solution corresponded to 1.94 ± 0.68 mg/mL, which represented a 1134-fold increase with respect to the pure drug (1.17 µg/mL) [35]. This value was significantly higher in comparison to other available solubility data from intravenous formulations, such as conjugates with polyvinyl alcohol (343 µg/mL) [9] and amphiphilic PVA-based micelles (111 µg/mL), and was comparable with those obtained for polymeric micelles made of amphiphilic dextrins (2.8 mg/mL) or branched polyethylene glycol (1.72 mg/mL) [7,8]. In addition, our formulation showed the highest DL in comparison to all the previously developed drug delivery systems, leading to better results with less polymer and no excipient or additive content. To assess the stability of the colloidal suspensions, different 4-HPR-P5 NP concentrations, ranging from 1 to 4 mg/mL, were kept in water in an incubator at 25 °C, and after three days the samples were microscopically inspected to detect the formation of precipitates and assayed for drug content in solution. No statistically significant differences were found in the period considered (SI, Figure S2). The preservation of supersaturation over time reflected the robustness of the interactions between the drugs and copolymers, which was also emphasized in the studies on drug release discussed below.

**Figure 6:**
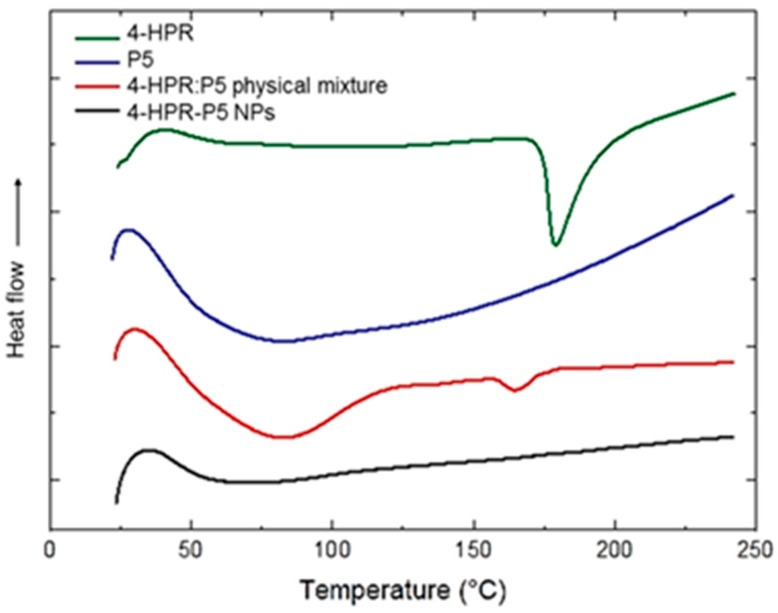
DSC thermograms of raw 4-HPR, P5, 4-HPR-P5 physical mixture, and 4-HPR-P5 NPs.

The study of the thermal properties of the drug and excipient mixtures allowed us to gain information about events such as melting, recrystallization, and decomposition, which in turn could help us assess the status of the entrapped drug (amorphized or crystallized) possibly modified during the antisolvent precipitation process. DSC thermograms of the yellow 4-HPR-P5 NP powder, in comparison to the corresponding physical mixture, as well as to raw 4-HPR and P5, are depicted in Figure 6. The 4-HPR thermogram showed the characteristic endothermic peak at 174.23 °C due to 4-HPR’s melting point [36]. P5 showed a broad endotherm corresponding to copolymer dehydration due to the numerous protonated amine groups, which made the material highly hydroscopic. The physical mixture profile confirmed the presence of the copolymer dehydration and the fairly evident melting peak of the drug. The 4-HPR-P5 NP profile appeared remarkably different, evidencing the disappearance of the 4-HPR melting peak, indicative of the absence of the drug in the crystalline state, which explained the great improvement in its solubility. Moreover, the broad initial peak also disappeared, suggesting that highly lipophilic drug molecules dispersed within the polymeric chains reduced P5’s tendency to adsorb humidity.

**Figure 7:**
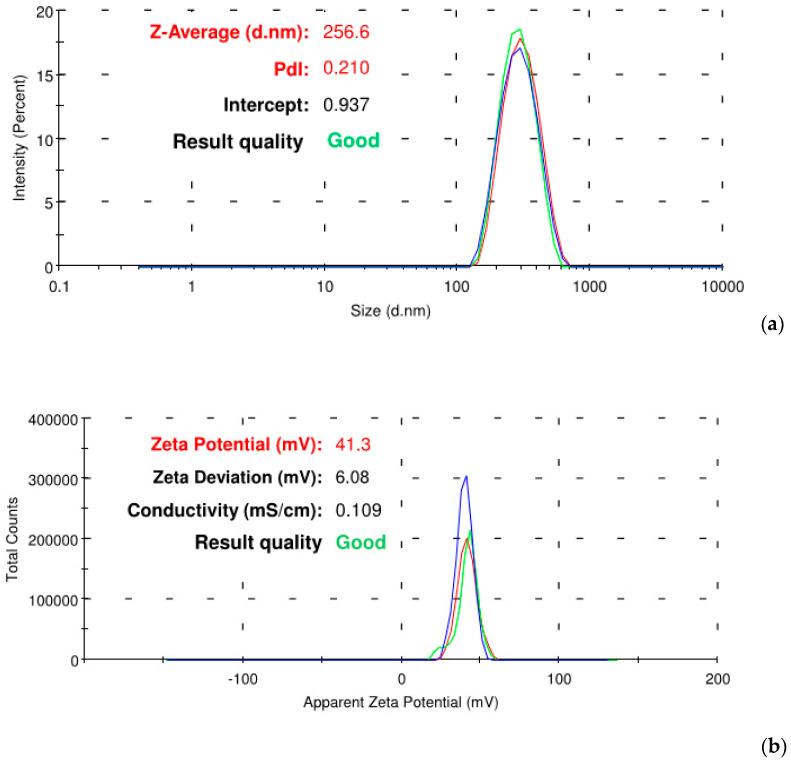
(**a**) Representative size distribution of 4-HPR-P5 NPs and (**b**) representative zeta potential obtained by dissolving 2 mg/mL of nanoparticles in water at 25 °C.

The mean particle sizes were measured after dissolving 2 mg/mL of 4-HPR-P5 NPs in water. The aim was to gain information on the suitability of 4-HPR-P5 in injectable formulations. In Figure 7, the representative size distributions and zeta potential are reported.

The loaded nanoparticles were characterized by a mean diameter of 249 ±10 nm and a narrow polydispersity of 0.227 ± 0.025. The particle size analysis revealed that the hydrodynamic mean diameter of the loaded NPs was lower than that of void P5 (334 ± 27 nm), which in addition was highly polydisperse (PDI = 1.012 ± 0.007) (SI, Figure S3) [24]. We could assume that the copolymer established loose and disorganized conformations in water, again because of the electrostatic repulsion between the protonated amine groups. On the contrary, when it was associated with the lipophilic molecules of the drug, the dehydration and the 4-HPR packing amongst the copolymer chains led to a more compact and organized supramolecular architecture. Similar behavior has already been noted in amphiphilic structures self-assembling in water [37]. As is well-known, nanoparticles should be able to circulate in the hematic flow for long enough to accumulate in the solid tumor by the enhanced permeability and retention effect (EPR). Size analysis confirmed the suitability of 4-HPR-P5 NPs to freely extravasate through the capillary discontinuity of the tumor tissue, thus improving the therapeutic efficacy of 4-HPR by the EPR effect. Additionally, the zeta potential of the 4-HPR-P5 NPs (ƺ = +41.3 ± 6.1 mV) was slightly less positive than that of P5 (ƺ = +57.6 ± 1.7 mV). The decrease in the positive charge on the surface of the copolymer had to be ascribed to the presence of the drug intimately associated to the P5, which induced a rearrangement of the spatial disposition of the polymeric chains.

### 2.5. In Vitro Release Profile of 4-HPR from 4-HPR-P5 NPs

**Figure 8:**
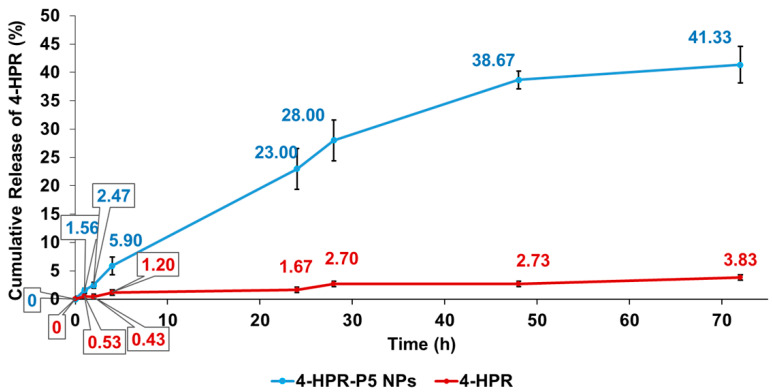
CR% of 4-HPR from NPs and from its suspension at pH 7.4, monitored for 72 h.

The experiments on the release of 4-HPR from 4-HPR-P5 NPs were carried out in PBS (pH = 7.4), and we performed the dialysis method as recently described [7]. The amount of free 4-HPR released from the nanoparticles was assessed by RP-HPLC using a previously constructed linear calibration curve available in SI (Figure S4). With the chromatographic conditions adopted, 4-HPR showed a retention time of 9.7605 ± 0.0782 min. The linearity of the method was good in the concentration range 1.03–20.60 μg/mL, with a correlation coefficient of 0.99119. The release profile highlighted a strong interaction between the copolymer and the drug, since the NPs had not delivered all their cargo in the time period considered. As depicted in Figure 8, sustained drug release was observed, with a fractional release at 48 h of 38.7% ± 1.5%. On the contrary, the amount of free 4-HPR present in the acceptor medium was extremely low, never reaching 4%, due to the low dissolution rate of the lipophilic drug molecules.

The amorphous drug dissolved much more rapidly than the crystalline form in aqueous solution due to the combined effects of the lack of a crystalline lattice and the elevated water solubility of the copolymer, leading to a supersaturated drug concentration. As shown in the stability studies, 4-HPR and P5 probably established strong interactions in solution that inhibited drug recrystallization. As already proposed in the case of polyvinylpyrrolidone [38], we assumed the presence of hydrogen bounding between the carbonyl groups of P5 and the phenol groups of 4-HPR. In addition, π–π interactions could have occurred between the aromatic rings, which would contribute to the stability of the complex and the enhancement of the drug apparent solubility. Nanoparticle-based delivery systems with high payloads and an extended release rate are especially promising to help create new therapeutic tools. Additionally, they can improve the delivery of currently used drugs by increasing the maximum tolerated dose (MTD), limiting the systemic distribution of cytotoxic agents, protecting against opsonization, decreasing clearance from the body, increasing dissolution rates, and localizing drugs by passive and/or active targeting [39]. As a result, the overall effect could be a significant improvement in drug bioavailability and therapeutic efficacy.

**Figure 9:**
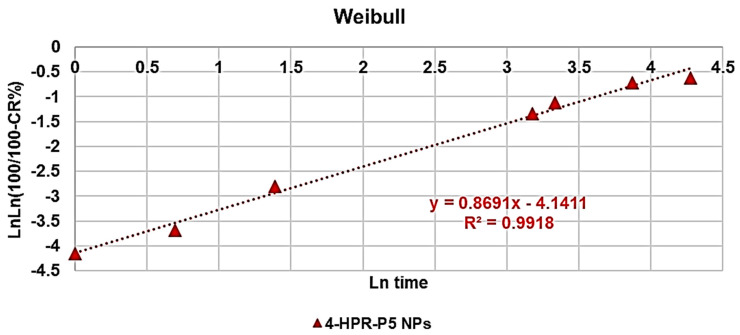
Linear regression of Weibull mathematical kinetic model with the related equation and R^2^ value.

The kinetics and the main mechanisms that govern the release of 4-HPR from 4-HPR-P5 NPs were determined by fitting the data of the 4-HPR release profile (cumulative release %, CR%) with the equations of zero-order, first-order, pseudo-second-order, Hixson–Crowell, Higuchi (or intra-particle diffusion), Weibull, and Korsmeyer–Peppas mathematical models [40]. The highest value of the coefficient of determination (R^2^) for the equations of the tendency lines related to the dispersion graphs obtained was considered as the parameter to determine which model best fit the release data. The R^2^ values were 0.9176 (zero-order), 0.9419 (first-order), 0.9587 (pseudo-second-order), 0.9883 (Korsmeyer–Peppas model), 0.9176 (Hixson–Crowell model), 0.9797 (Higuchi model), and 0.9918 (Weibull model), establishing that the release of 4-HPR from the developed delivery system best fit with the Weibull mathematical model (Figure 9).

The Weibull kinetic model is given by the following Equation (1):

(1) $$LnLn\frac{C_{0}}{C_{0}-C_{t}}=\beta Lnt+Ln\alpha \;\;\;$$

where *C_t_* is the concentration of drug release at time *t*, *C*_0_ is the initial concentration of drug present in the nanocomposite system, *t* is the time, *β* is the shape parameter of the dissolution curve, and α is the scale parameter. According to the linear regression shown in Figure 9 and Equation (1), the slope of the regression corresponds to the value of *β*, while the value of *α* can be calculated by the value of the intercept (*Ln α*). A value of *β* = 1 indicates a first-order release, in which the drug concentration gradient in the dissolution medium controls the rate of its release, while *β* > 1 indicates that a complex mechanism governs the release process. Values of *β* < 0.75 imply that the drug release is governed by diffusion mechanisms, while values in the range 0.75–1.0, as in the present case, suggest a combined mechanism, which is frequently encountered in release studies [41].

Conversely, the R^2^ values for the release of non-formulated 4-HPR were 0.9008 (zero-order), 0.9029 (first-order), 0.8635 (pseudo-second-order), 0.9155 (Korsmeyer–Peppas model), 0.9008 (Hixson–Crowell model), 0.9513 (Higuchi model), and 0.9158 (Weibull model), establishing that, as expected, the release of free 4-HPR best fit with the Higuchi mathematical model and was therefore dependent on Fickian diffusion (Figure S5).

### 2.6. Cytotoxic Activity of 4-HPR and 4-HPR-P5 NPs on Neuroblastoma Cell Lines

**Figure 10:**
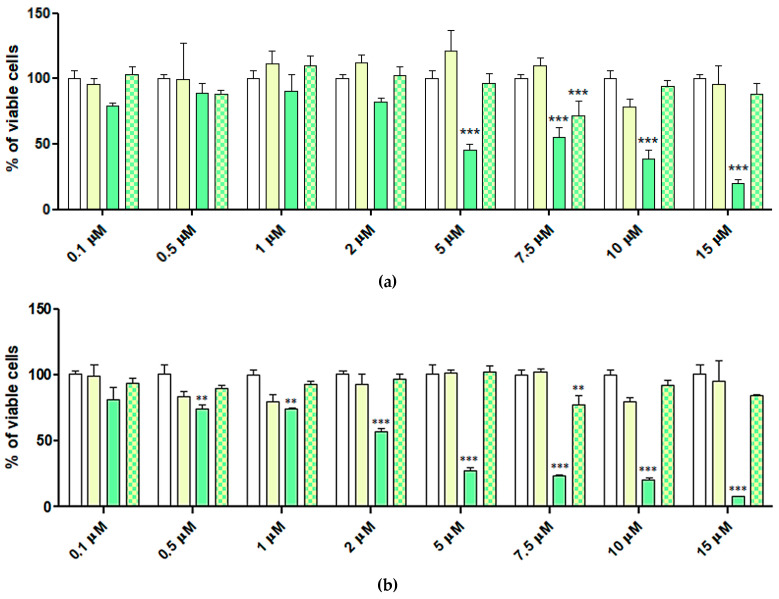

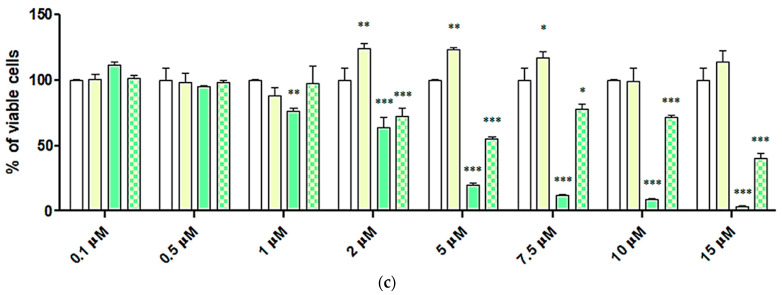
Dose- and time-dependent cytotoxicity activity of P5, 4-HPR, and 4-HPR-P5 NPs at 24 h (**a**), 48 h (**b**), and 72 h (**c**) in SH-SY5Y cells. Concentration values reported on the x-axis refer to 4-HPR, while the concentrations used for P5 and 4-HPR-P5 NPs can be derived from Table 2. White columns: control; yellow columns: P5; green columns: free 4-HPR; and checkered columns: 4-HPR-P5 NPs. Significance refers to control (*p* > 0.05 ns; *p* < 0.05 *; *p* < 0.01 **; *p* < 0.001 ***).

**Figure 11:**
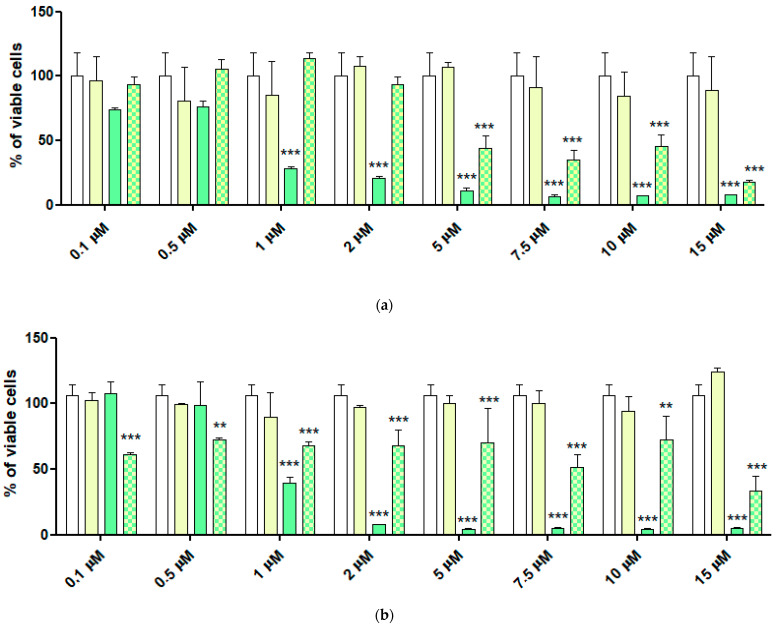

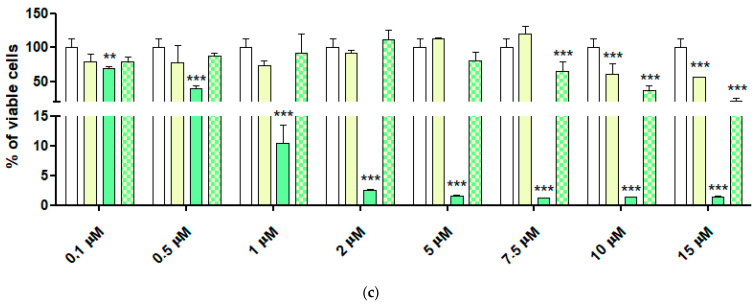
Dose- and time-dependent cytotoxicity activity of P5, 4-HPR, and 4-HPR-P5 NPs at 24 h (**a**), 48 h (**b**), and 72 h (**c**) in IMR-32 cells. Concentration values reported on the x-axis refer to 4-HPR, while the concentrations used for P5 and 4-HPR-P5 NPs can be derived from Table 2. White columns: control; yellow columns: P5; green columns: free 4-HPR; and checkered columns: 4-HPR-P5 NPs. Significance refers to control (*p* > 0.05 ns; *p* < 0.01 **; *p* < 0.001 ***).

Dose- and time-dependent cytotoxicity experiments were performed to evaluate the effects of HPR-P5 NPs on IMR-32 and SH-SY5Y neuroblastoma (NB) cells. We selected these cell lines as representative of genetically male and female NB cell lines, respectively. NB cells were exposed to increasing concentrations of pristine HPR for 24, 48, and 72 h. At the same time, cells were treated with 4-HPR-P5 concentrations providing the same amount of drug, according to the DL%. In addition, from the DL value and the molecular weights (MWs) of P5 and 4-HPR, we could obtain the number of 4-HPR moles that were loaded per P5 mole, which was 7.6 [20,26,41]. This number was helpful to obtain the MW of the complex 4-HPR-P5, which was 8075.4 [26,29,41]. Using these data, we found the concentrations of HPR-P5 NPs necessary to provide the same concentrations of 4-HPR utilized. NB cells were also treated with P5 alone at the concentrations provided by HPR-P5 NPs. The aim was to assess the cytotoxicity of the cationic macromolecule (copolymer P5) used to entrap 4-HPR, and to prove whether the nanotechnological manipulation of 4-HPR using P5, in addition to enhancing its water solubility, succeeded in improving its cytotoxic effects. The concentrations of each sample administered to the cells are detailed in Table 2, while the results obtained against the SH-SY5Y and IMR-32 NB cells are reported in Figure 10 and Figure 11, respectively.

**Figure 12:**
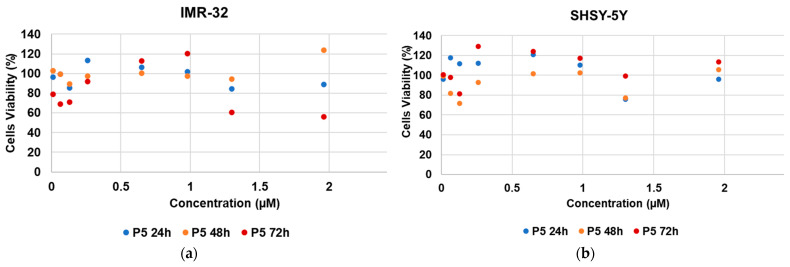
Dose-dependent cytotoxicity activity of P5 at 24 h (blue), 48 h (orange), and 72 h (red) in IMR-32 cells (**a**) and SH-SY5Y cells (**b**) in the range 0.013–1.96 µM.

**Figure 13:**
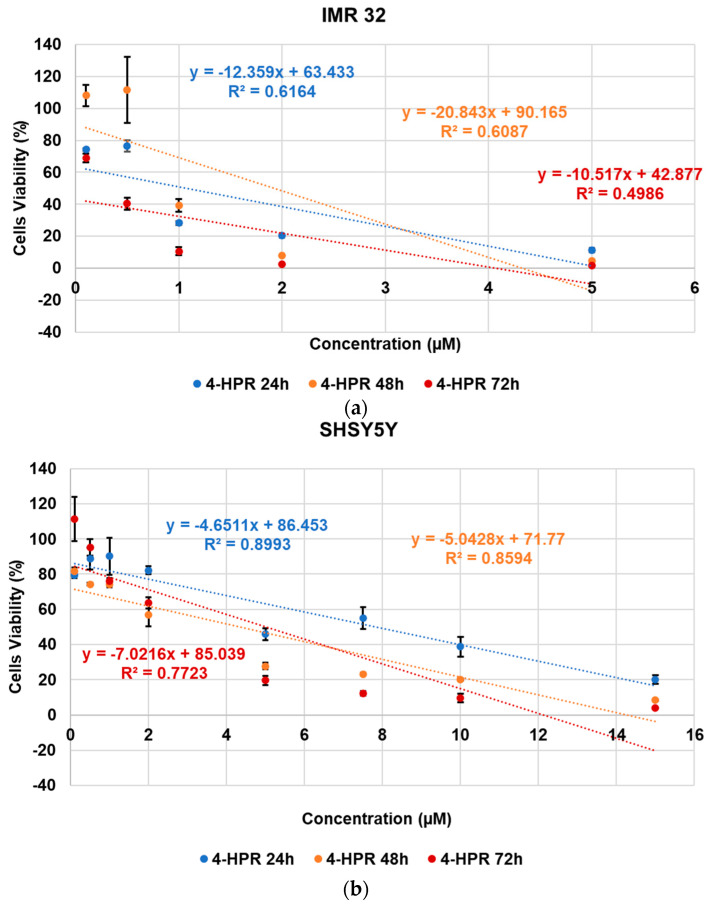
Dose-dependent cytotoxicity activity of 4-HPR at 24 h (blue), 48 h (orange), and 72 h (red) in IMR-32 cells (**a**) and SH-SY5Y cells (**b**) in the range 0.1–5 µM (**a**) and 0.1-15 µM (**b**).

**Figure 14:**
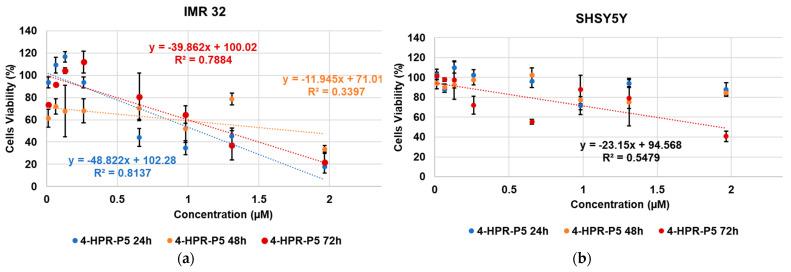
Dose-dependent cytotoxicity activity of 4-HPR-P5 NPs at 24 h (blue), 48 h (orange), and 72 h (red) in IMR-32 cells (**a**) and SH-SY5Y cells (**b**) in the range 0.013–1.96 µM.

P5 did not cause a significant reduction in the viability of IMR-32 NB cells at all concentrations tested, except for prolonged exposure (72 h), when administered at concentrations ≥ 1.30 µM in a dose-dependent way. The viable cells accounted for >50% at all concentrations tested and, in some cases, proliferation was observed. In this regard, by combining P5 with 4-HPR, its cytotoxicity was significantly improved. Indeed, when exposed to 4-HPR-P5 NPs, the viable cells accounted for <50% at a concentration of 0.66 µM after 24 h of exposure, at 1.96 µM after 48 h, and at 1.31 µM after 72 h, thus establishing that the cytotoxicity of the complex did not depend on the time of exposure for this cell line. Specifically, while free 4-HPR determined significant reductions in cell viability for concentrations ≥ 1 µM (24 and 48 h) and ≥0.1 µM (72 h), 4-HPR-P5 NPs were significantly cytotoxic at concentrations ≥ 0.66, 0.013, and 0.98 µM at 24, 48, and 72 h, respectively. On SH-SY5Y cells, P5 was even less effective than on IMR-32 cells, since a significant reduction in proliferation was not observed, while after a longer duration of exposure an increment in the number of viable cells was detected. Free 4-HPR determined a significant reduction in cell viability depending on the time of exposure, like 4-HPR-P5 NPs, which showed cytotoxic activity over time, particularly at concentrations ≥ 0.26 µM. Nevertheless, considerable fluctuations in the percentage of viable cells were still detected for higher concentrations. To better compare the cytotoxic effects of 4-HPR, P5, and 4-HPR-P5 NPs and to assess if the formulation of 4-HPR in NPs using P5 had a positive impact on the cytotoxic effects of 4-HPR on NB cells, we plotted the viable cells (%) vs. concentrations for all samples, obtaining the dispersion graphs reported in Figure 12, Figure 13 and Figure 14. Then, using the equations of the linear regressions associated with the dispersion graphs obtained, we found the IC50 for all samples, which are reported in Table 3.

As reported above, Figure 12a,b, showing the cytotoxic profile of P5, confirmed that in the range of concentrations considered, cell viability remained over 50% and proliferation was observed regardless of the increasing concentrations and prolonged times of exposure, especially for SH-SY5Y cells. In this regard, it was not possible to calculate the values of IC50 for P5.

The 4-HPR showed cytotoxic effects that were especially dependent on the dose and less dependent on the exposure time, and IMR-32 cells were more susceptible than SH-SY5Y cells. In the case of 4-HPR, the highest cytotoxicity occurred after 24 and 72 h of exposure, with the IC50 values being 1.08 and 0.68 µM, respectively, vs. the IC50 = 1.93 µM calculated at 48 h. Towards SH-SY5Y cells, 4-HPR was 7.2-fold less cytotoxic than towards IMR-32 cells at 24 h, and a lower IC50 was observed after 48 h of exposure (4.32 µM).

Like 4-HPR, 4-HPR-P5 NPs were also more cytotoxic against IMR-32 cells than SH-SY5Y cells, as the viability of the latter remained over 50% after 24 and 48 h of exposure, while the IC50 calculated at 72 h was 1.93 µM and 2.6-fold lower than that computed for 4-HPR in the same conditions. For IMR-32 cells, 4-HPR-P5 NPs showed cytotoxic effects like those exerted by 4-HPR at both 24 and 48 h (IC50 = 1.07 vs. 1.08 µM, 1.76 vs. 1.93 µM, respectively), and to a lesser extent at 72 h (IC50 = 1.25 vs. 0.68 µM).

These results were overall in agreement with in vitro release studies; indeed, the delivery of 4-HPR from the NPs took place in a gradual manner, and so more time was needed for the drug to exert its effect. To perform biological studies, free 4-HPR was dissolved in ethanol and then diluted in fetal bovine serum (FBS) and finally in complete medium, since the cells could not be exposed to the drug suspension, as the crystals could cause physical damage to the cells. In these conditions, free 4-HPR could trigger its cytotoxic effects in a shorter time with respect to complexed 4-HPR, which obviously needed more time to be delivered. It should be noted that the timing of release is of paramount importance in vivo, where the early leakage of the drug may nullify the benefits obtained when associated with the carrier. The great advantage of the formulation is the ability to avoid the use of organic solvents, but at the same time achieve high drug concentrations, which can be exploited for both oral and injectable dosage forms. Moreover, carrier-assisted drug delivery systems have been widely used to treat cancer due to their ability to enhance the biological stability and bioavailability of drugs. In particular, they are able to significantly reduce undesired side effects by achieving the site-specific delivery of chemotherapeutics through the EPR effect. Based on these considerations, our future plans include studying the therapeutic potential of our formulation in more depth by conducting preclinical studies on a NB mouse model.

## 3. Materials and Methods

### 3.1. Experimental Procedure to Prepare 4-HPR-Loaded P5-Based NPs

The 4-HPR-P5 NPs were prepared by the nanoprecipitation technique [26], which was selected from several processing approaches to prepare amorphous materials, including hot-melt extrusion and spray drying [17,18,19,20,21,22,42]. Briefly, a methanol (MeOH) 1/1 wt/wt solution of P5 and 4-HRP was prepared. Specifically, P5 (104.6 mg, 0.0205 mmol) and 4-HPR (101.5 mg, 0.2593 mmol, 12.6 equivalents) were dissolved in 3 mL MeOH and sonicated (20 min.) to maximize polymer–drug interactions, producing a clear yellow solution. Diethyl ether (Et_2_O = 30 mL) was used as a non-solvent. The yellow solution of the two ingredients (P5 and 4-HPR) was added to the non-solvent phase drop-wise using a Pasteur at room temperature and under moderate magnetic stirring (500 rpm). A fine dispersion was obtained, which was promptly centrifuged at 4000 rpm for 20 min. No surfactant was added during the antisolvent co-precipitation process, thus achieving an ASD characteristic of the second-generation SDs [23]. Once the turbid supernatant (1S) was separated, the solid was resuspended in Et_2_O; the suspension was sonicated for 10 min at 25 °C and centrifuged again at 4000 rpm for 20 min; and the precipitated yellow solid (1P) was separated from the solvents. Meanwhile, the supernatant 1S was evaporated at a reduced pressure using a Rotavapor^®^ R-3000 (Büchi Labortechnik, Flawil, St. Gallen, Switzerland), and the obtained yellow oil was treated with Et_2_O to obtain further yellow precipitate (2P). The suspension of 2P in Et_2_O was added to 1P; further Et_2_O was added; and the final dispersion was sonicated (10 min, room temperature) and then centrifuged (4000 rpm, 20 min). The final solid was separated from the solvent, and it was brought to a constant weight at a reduced pressure, producing 163.8 mg of an amorphous yellow solid, which was stored in a freezer in the dark to avoid the degradation of the 4-HPR. Subsequently, additional complex 4-HPR-P5 (8.4 mg) was precipitated from the organic solvents and stored separately from the first precipitate. The evaporation of the collected organic solvents allowed us to recover the unentrapped 4-HPR, as confirmed by TLC (AcOEt/n-hexane ¼, Rf. 0.16); FTIR analyses (Figure 1); and PCA (Figure 2).

FTIR (KBr, ν, cm^−1^): 3450 (OH of 4-HPR and NH3+ of P5), 2926 (stretching CH alkyls of 4-HPR and P5), and 1617 (C=O amides of 4-HPR and P5).

### 3.2. Chemometric-Assisted ATR-FTIR Spectroscopy

FTIR spectra of 4-HPR, recovered 4-HPR (R-4-HPR), P5, and 4-HPR-P5 NPs were recorded in triplicate on samples in the form of KBr pellets in transmission mode using a Spectrum Two FT-IR Spectrometer (PerkinElmer, Inc., Waltham, MA, USA). Acquisitions were obtained from 4000 to 600 cm^−1^, with a 1 cm^−1^ spectral resolution; the co-addition of 32 interferograms; and a measurement accuracy in the frequency data at each measured point of 0.01 cm^−1^, due to the laser internal reference of the instrument. The frequency of each band was obtained automatically using the ‘‘find peaks’’ command of the instrument software. The matrix of the spectral data was subjected to PCA by means of CAT statistical software (Chemometric Agile Tool, freely downloadable online, at: http://www.gruppochemiometria.it/index.php/software/19-download-the-r-based-chemometric-software; accessed on 1 February 2023). We organized the FTIR datasets of all the spectra acquired in a matrix of 3401 × 4 (n = 13604) measurable variables. For each sample, the variables consisted of the values of transmission (%) associated with the wavenumbers (3401) in the range 4000–600 cm^−1^. The system was simplified via PCA, which is a chemometric tool able to reduce a large number of variables to a small number of new variables, namely principal components (PCs) [29,30].

### 3.3. Potentiometric Titration of 4-HPR-P5 NPs

Potentiometric titrations were performed on 4-HPR-P5 NPs at room temperature, and their titration curve was obtained. In a representative experiment, an exactly weighted sample of 4-HPR-P5 (14.8 mg) was dissolved in 20 mL of Milli-Q water (m-Q) and a pH = 7.41 was measured. The obtained yellow solution was treated under magnetic stirring with a standard 0.1 N NaOH aqueous solution (2.0 mL, pH = 10.59). The solution was potentiometrically titrated under stirring by adding aliquots of HCl 0.1N up to pH = 3, for a total volume of 5.0 mL [27,28]. Titrations were performed in triplicate, and measurements were reported as mean ± S.D. The titration curve shown in the Discussion section is that generated by plotting the data obtained by carrying out the representative experiment described here.

### 3.4. Characterization of 4-HPR-P5 NPs: Drug Loading, Solubility, and Stability

To assess the drug loading content % (DL%) of solid 4-HPR-P5 NPs, the yellow powder obtained from nanoprecipitation was accurately weighed and dissolved in MeOH to induce the dissociation of the drug from P5. The concentration of 4-HPR was determined by UV-Vis spectrophotometric analysis at λ_max_ = 364 nm using a UV-Vis spectrophotometer (HP 8453, Hewlett Packard, Palo Alto, CA, USA). The drug content in the samples was evaluated on the basis of a calibration curve obtained by measuring the absorbance of standard solutions of 4-HPR in the same solvent (Figure S1). The DL% was calculated according to the formula in Equation (2). All experiments were carried out in triplicate, and the results are reported as mean ± standard deviation (S.D.).

(2) $$\mathrm{DL}\%=\frac{\mathrm{wheight}\;\mathrm{of}\;\mathrm{drug}\;\mathrm{in}\;\mathrm{the}\;\mathrm{loaded}\;\mathrm{NPs}}{\mathrm{wheight}\;\mathrm{of}\;\mathrm{the}\;\mathrm{solid}\;\mathrm{NPs}}\times 100$$

The aqueous solubility of solid 4-HPR-P5 NPs was assessed by adding increasing amounts of powder to 10 mL of water in a sealed vial until a precipitate was obtained and visually inspected by an optical microscope. Replicated samples were maintained at 25 °C under stirring in a WTB © BINDER GmbH 2015–2020 incubator (Im Mittleren Ösch 5, D-78532 Tuttlingen, Germany) and filtered after 24 h using a 0.22 µm filter (Minisart RC Sartorius, GER). Aliquots of each filtrate were diluted with methanol and spectrophotometrically analyzed up to an absorbance plateau indicative of water saturation. Samples without the drug were prepared as blanks. The stability of three supersaturated 4-HPR-P5 NP solutions (1,2,4 mg/mL) was investigated by storing the colloidal dispersions in the liquid state at 25 °C in the incubator and visually observing them after 24, 48, and 72 h for signs of precipitation. At each time point, the drug concentration in solution was determined after filtration to remove the 4-HPR that eventually precipitated. The determinations were made in triplicate, and the results are reported as mean ± SD.

### 3.5. Differential Scanning Calorimetry (DSC)

To confirm the entrapment of 4-HPR inside the nanoparticles, DSC analysis was performed. The thermal properties of the yellow 4-HPR-P5 NP powder obtained from nanoprecipitation, free 4-HPR and P5, and the physical mixtures of the raw materials in equal ratios to that of the prepared 4-HPR-P5 NPs were studied using a Discovery SDT 650 equipped with TRIOS software (TA Instrument, New Castle, DE, USA). The instrument was calibrated with sapphire and zinc, and about 4 mg of each sample was crimped in alumina pans. The thermograms were recorded from 25 to 250 °C at a heating rate of 10 °C/min under nitrogen flow.

### 3.6. Determination of Size, Polydispersity Index, and Zeta Potential

The particle size (Z-average), polydispersity index (PDI), and zeta potential (ƺ) of the colloidal suspensions were measured at 25 °C using a Malvern Nano ZS90 light-scattering apparatus (Malvern Instruments Ltd., Worcestershire, UK) at a scattering angle of 90°. The apparent equivalent hydrodynamic *radii* of the NPs were calculated using the Stokes–Einstein equation. The ƺ potential values of micelles were recorded with the same apparatus in distilled water at 25 °C. The results from these light-scattering experiments are presented as the average values ± SD obtained from three different batches after carrying out three runs of ten measurements per sample.

### 3.7. In Vitro Release Studies

The in vitro studies of drug release from the 4-HPR-P5 NPs were carried out as previously reported [7]. Briefly, an amount of 4-HPR-P5 NP powder corresponding to 2 mg of 4-HPR was reconstituted with 5 mL of phosphate buffer solution (PBS) at pH 7.4. The colloidal suspension was placed in a dialysis tube (CE Dialysis Tubing MW CO 100–500 Da, SpectrumTM, Spectra/Pore^®^, Thermo Fischer Scientific, Waltham, MA, USA), allowing diffusion only for the free drug, and then dialyzed against 50 mL of isotonic PBS at pH 7.4 and 10 mL of chloroform. Following the report in [43], an organic solvent (chloroform in our case) was added to the aqueous medium to form the biphasic dissolution model (BDM) first proposed by Levy [44]. In particular, the BDM assured sink conditions throughout the experiment, providing the continuous extraction of the drug diffused through the membrane. In the BDM, the presence of an organic phase within the dissolution medium could act as a reservoir for the dissolved drug [43]. Crucially, the aqueous layer does not saturate, sink conditions are maintained, and the experiment will, in theory, yield complete dissolution [43]. A corresponding 4-HPR raw powder was suspended in the same volume of solution to obtain an equal drug concentration and tested along with the loaded NPs. The system was thermostated at 37 ± 0.5 °C, and each time point was tested in triplicate. At fixed time intervals, chloroform was removed and evaporated, and the residue was dissolved in 300 μL of acetonitrile. The resulting solutions were analyzed by RP-HPLC DAD to determine the amount of drug released over time. The results are expressed as 4-HPR cumulative release percentages (CR %), which were calculated for each time point by Equation (3):

(3) $$\mathrm{CR}\;\left(\%\right)=\frac{4-\mathrm{HPR}\;\left(t\right)}{4-\mathrm{HPR}\;\left(i\right)}\times 100\;$$

where 4-HPR (t) is the amount of 4-HPR released at incubation time *t*, while 4-HPR (i) is the total 4-HPR loaded in the dialysis tube or the total 4-HPR entrapped in the weight of 4-HPR-P5 NPs analyzed according to the computed DL%.

The HPLC analysis of 4-HPR was performed by a Hewlett-Packard HP1100 HPLC system (Palo Alto, CA, USA) consisting of a quaternary pump and a continuous vacuum degasser equipped with a Rheodyne 7125 manual sample injector and a Hewlett-Packard HP UV–vis diode array detector (DAD). Briefly, chromatographic separations were achieved by a LiChroCART Purospher Star RP18-e column (250 mm × 4.6 mm i.d.) (5 µm) (Merck, Darmstad, Germany) combined with a Merck LiChroCART 4-4 LiChrospher 100 RP18 (5 µm) guard column using an isocratic elution with a mobile phase of CH_3_CN:H_2_O:CH_3_COOH (80:18:2, *v*/*v*/*v*), flow rate 1 mL/min, absorbance detector set at 360 nm, and injection volume of 20 µL [18,19]. A HP ChemStation data system was used for data acquisition and handling. The limit of detection of 4-HPR was 15 ng/mL. The determinations were made in triplicate, and the results are expressed as mean ± SD.

### 3.8. Cell Viability Studies

The human neuroblastoma cell lines IMR-32 and SH-SY5Y were maintained in complete medium (Dulbecco’s modified Eagle medium; Sigma) containing 10% *v*/*v* heat-inactivated fetal bovine serum (Gibco-Invitrogen S.r.l., Carlsbad, CA, USA) and 50 IU/mL penicillin G; 50 μg/mL streptomycin sulphate; and 2 mM L-glutamine (all reagents from Euroclone S.p.A., Milan, Italy). Cells were periodically tested for mycoplasma contamination (Mycoplasma Reagent Set, Aurogene s.p.a, Pavia, Italy). To assay cell proliferation under 4-HPR exposure, cells were seeded in triplicate in a 96w plate at 3000 to 10,000 cells per well in 200 μL of complete medium. After 24 h, the medium was changed, and the cells were exposed for 24, 48, or 72 h to free 4-HPR at 0.1, 0.5, 1, 2, 5, 7.5, 10, and 15 µM; 4-HPR-P5 NPs at concentrations able to provide the same concentrations of free 4-HPR; and P5 at the concentrations provided by the amounts of 4-HPR-P5 NPs tested. The effect on cell growth was evaluated by a fluorescence-based proliferation and cytotoxicity assay (CyQUANT^®^ Direct Cell Proliferation Assay, Thermo Fisher Scientific, Life Technologies, MB, Italy) according to the manufacturer’s instructions. Briefly, at the selected times, an equal volume of detection reagent was added to the cells in culture and incubated for 60 min at 37 °C. The fluorescence of the samples was measured using the monochromator-based M200 plate reader (Tecan, Männedorf, Switzerland) set at 480/535 nm.

### 3.9. Statistical Analysis

All the experiments were performed at least three times. Each set of experimental conditions, for the biological assays, was tested in 96-well plates and carried out in triplicate. Differential findings among the experimental groups were determined by two-way ANOVA (analysis of variance) with Bonferroni post-tests using GraphPad Prism 5 (GraphPad Software v5.0, San Diego, CA, USA). Asterisks indicate the following *p*-value ranges: * = *p* < 0.05, ** = *p* < 0.01, *** = *p* < 0.001.

## 4. Conclusions

Despite the several technological approaches that have been developed over the last two decades, no effective formulation of Fenretinide is currently commercially available. In this context, herein we proposed the preparation of an ASD as a solubilizing technique, taking advantage of a hydrophilic copolymer previously synthesized by our team. This approach is quite simple, low-cost, and easily up-scalable compared to more complex techniques such as spray drying or freeze drying, as evidenced by the increase in the number of marketed products approved by the US Food and Drug Administration.

A powder formulation of Fenretinide molecularly dispersed and entrapped within the hydrophilic scaffold of P5 was achieved by employing the antisolvent co-precipitation technique. We used MeOH as a solvent and Et_2_O as an antisolvent, as they both have boiling points that allowed the easy isolation of the product without its exposure to high temperatures. The amorphization was confirmed by thermal studies, and the high payload reached determined an increase in the drug apparent solubility of 1134 folds. While this remarkable enhancement in the drug concentration in water may be useful for the future preparation of injectable formulations, the nanometric dimension of the NPs may be exploited for their accumulation in tumor tissue. In addition, the supersaturated drug solution generated by the dissolution of the 4-HPR-loaded NPs could increase the oral absorption of 4-HPR according to the Noyes–Whitney law. The in vitro antiproliferative activity observed for 4-HPR-P5 was in agreement with the slow release of the drug from the formulation, while the calculated IC50 values were comparable or even lower than those of free 4-HPR.

Since during shelf-life studies, amorphous solid dispersions may undergo solid-state physical instability associated with drug recrystallization, the preservation of the amorphous form is currently under investigation. If some recrystallization is detected, other more expensive amorphization techniques, such as spray drying or freeze drying, using the same polymer–solvent system will be considered.

## Figures and Tables

**Table 1:** Maximum β value and β_AVE_ of 4-HPR-P5 NPs, P5 alone, and commercial polyethyleneimine (PEI-*b*) in the pH range 4.5–7.5 computed from potentiometric titration data.

Entry	β (pH Value)	β_AVE_ * (mL/pH)
P5	0.667 (6.10)	0.2305 ± 0.1354
4-HPR-P5 NPs	1.92 (6.62)	0.6167 ± 0.3468
PEI-*b*	0.08261 (7.33) 0.0760 (6.81)	0.517 ± 0.2541

* Defined as the volume of HCl 0.1N necessary to decrease the pH by one unit in the pH range 4.5–7.5, reported as mean ± SD (*n* = 3).

**Table 2:** Concentrations of each sample administered to NB cells.

Samples	Concentrations (µM)							
HPR	0.1	0.5	1	2	5	7.5	10	15
HPR-P5	0.0132	0.0655	0.1310	0.2620	0.6551	0.9827	1.3100	1.9654
P5	0.0130	0.0650	0.1300	0.2600	0.6500	0.9803	1.3000	1.9606

**Table 3:** IC50 of 4-HPR, P5, and 4-HPR-P5 NPs for IMR-32 and SH-SY5Y NB cells at 24, 48, and 72 h.

Cells	Times (h)	4-HPR (µM)	P5 (µM)	4-HPR-P5 NPs (µM)
IMR-32	24	1.08	N.D.	1.07
	48	1.93	N.D.	1.76
	72	0.68	N.D.	1.25
SH-SY5Y	24	7.84	N.D.	N.D.
	48	4.32	N.D.	N.D.
	72	4.99	N.D.	1.93

N.D. = not detected.

## Data Availability

All data concerning this study are contained in the present manuscript or in previous articles whose references have been provided.

## Supplementary Materials

The following supporting information can be downloaded at: https://www.mdpi.com/article/10.3390/ph16030388/s1.
