# Supplementary material for: Preparation and Characterization of Amorphous Solid Dispersions for the Solubilization of Fenretinide
Source: Pharmaceuticals (Basel). 2023 Mar 2;16(3):388. doi: 10.3390/ph16030388 (PMC10052200; doi:10.3390/ph16030388)
Supplement: Supplementary file 1 [file pharmaceuticals-16-00388-s001.zip › pharmaceuticals-2227806-supplementary.pdf]

pharmaceuticals

Supplementary Materials

Preparation and Characterization of Amorphous Solid Dispersions for the Solubilization of Fenretinide

Guendalina Zuccari [1,*], Eleonora Russo [1], Carla Villa [1], Alessia Zorzoli [2], Danilo Marimpietri [2], Leonardo Marchitto [3], and Silvana Alfei [1]

[1] Department of Pharmacy (DiFAR), University of Genoa, Viale Cembrano 4, I-16148, Genova, Italy,
[2] Cell Factory, IRCCS Istituto Giannina Gaslini, Via Gerolamo Gaslini 5, I-16147 Genova,
[3] Department of Sciences for the Quality of Life, University of Bologna, Corso D'Augusto 237, I-47921 Rimini, Italy
* Correspondance: guendalina.zuccari@unige.it

Section S1. Figure S1-S5.

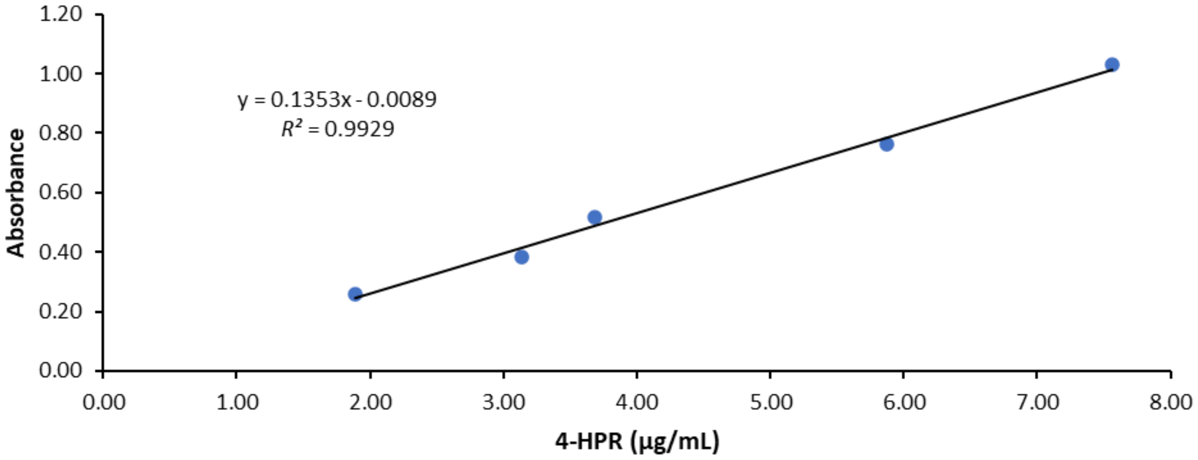

Figure S1. 4-HPR calibration curve in methanol at λmax = 364 nm using an UV-Vis spectrophotometer.

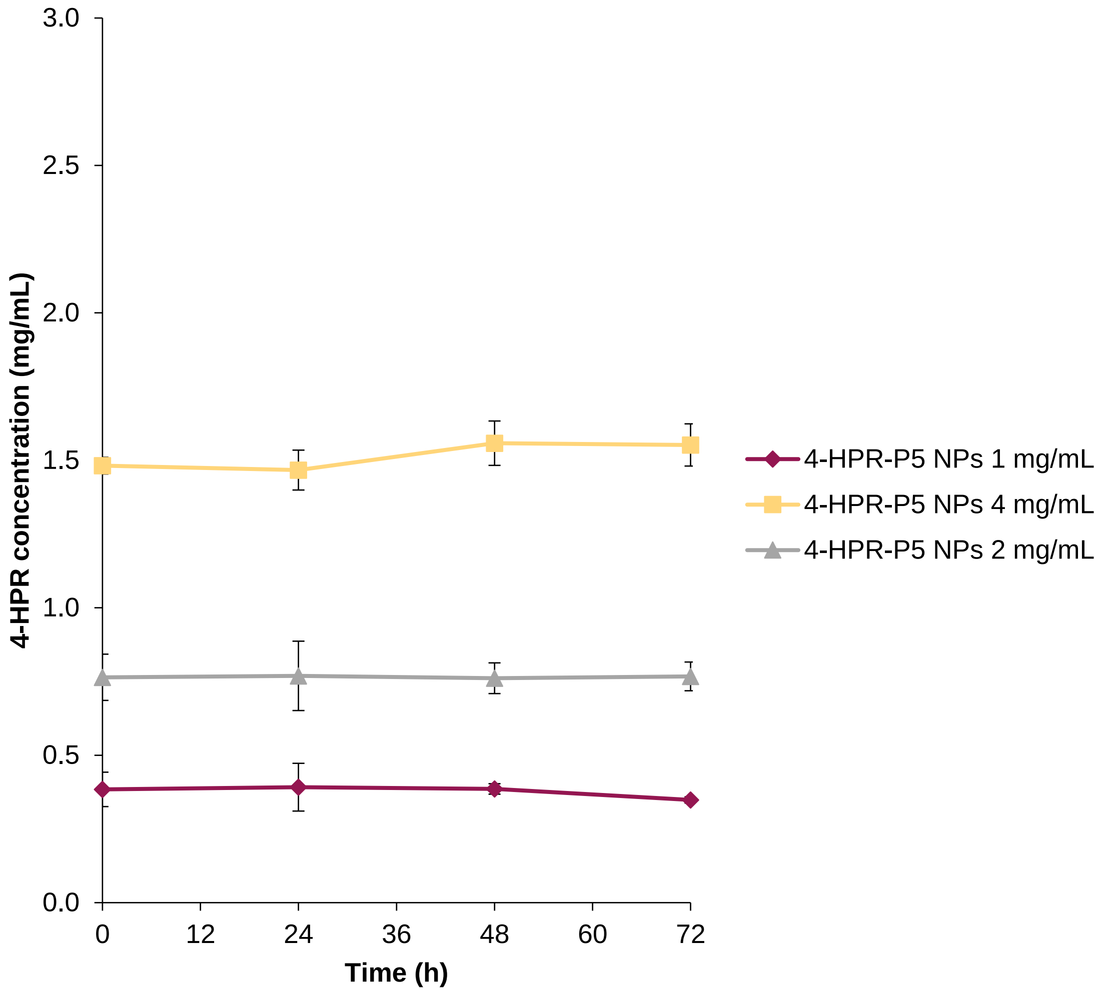

Figure S2. Stability over time of aqueous nanoparticle dispersions at different concentrations (1,2,4 mg/mL) maintained at 25 °C. Data of the apparent 4-HPR solubility over time are reported as the mean ± standard deviation (S.D.) of three independent experiments.

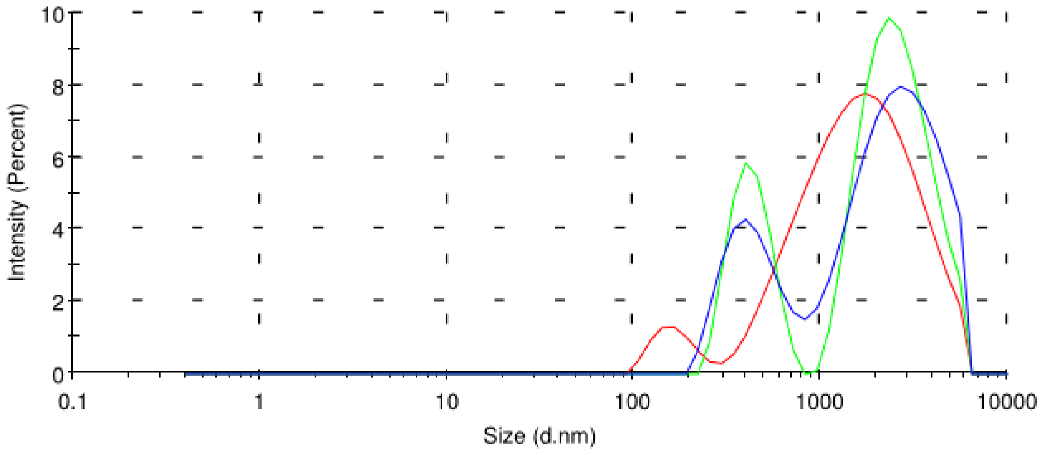

Figure S3. Representative size distribution of P5 recorded in water at 25°C.

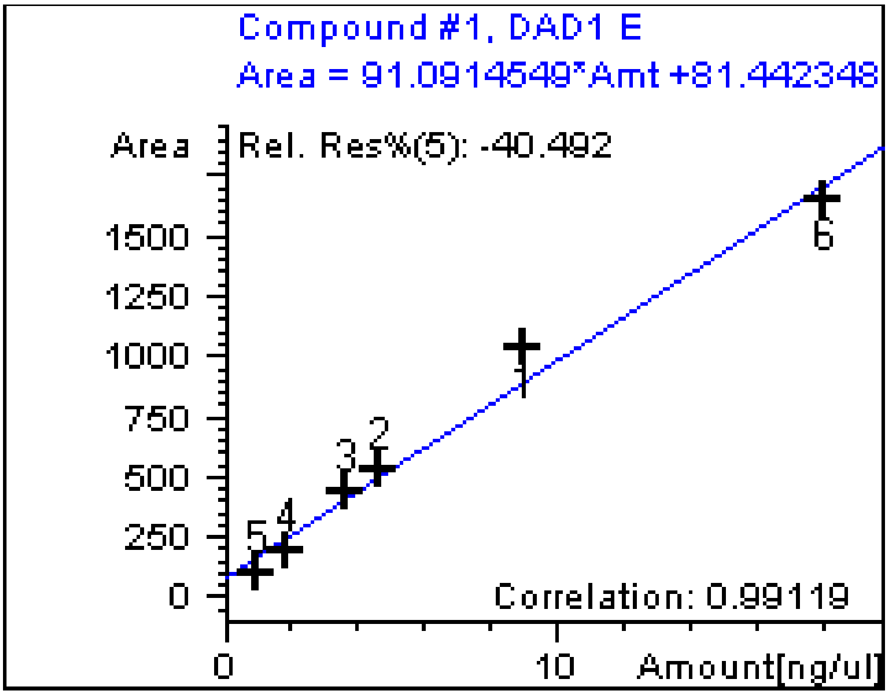

Figure S4. 4-HPR calibration curve using RP-HPLC DAD C_{18} (5 µm) reverse-phase column (150 mm × 4.6 mm). Mobile phase: isocratic elution with acetonitrile/water/glacial acetic acid (80/18/2 v/v/v) delivered at 1 mL/min. Absorbance detector set at λ_{max} = 260 nm.

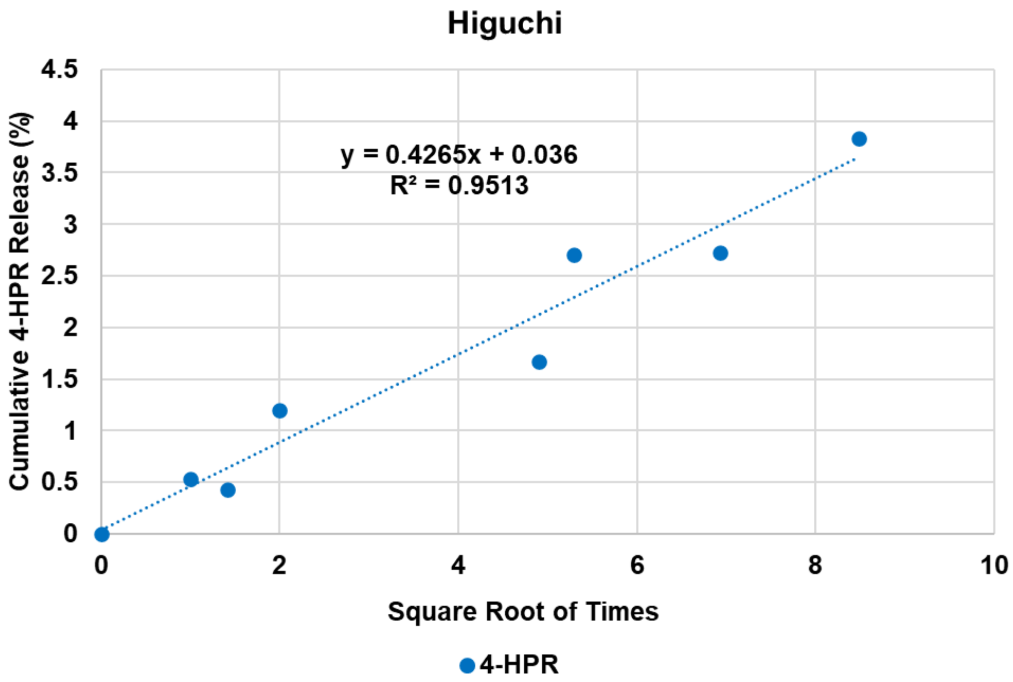

Figure S5. Linear regression of Higuchi mathematical kinetic model with the related equation and R^2 value.
